# Gut Microbiota and Dysbiosis in Alzheimer’s Disease: Implications for Pathogenesis and Treatment

**DOI:** 10.1007/s12035-020-02073-3

**Published:** 2020-08-23

**Authors:** Shan Liu, Jiguo Gao, Mingqin Zhu, Kangding Liu, Hong-Liang Zhang

**Affiliations:** 1 Department of Neurology, First Hospital of Jilin University, Jilin University, Xinmin Street 71, Changchun, 130021 China; 2 Departments of Laboratory Medicine and Pathology, Neurology and Immunology, Mayo Clinic, Rochester, MN USA; 3 Department of Life Sciences, National Natural Science Foundation of China, Shuangqing Road 83, Beijing, 100085 China

**Keywords:** Alzheimer’s disease, Gut microbiota, Microbiota-gut-brain axis, Gut dysbiosis

## Abstract

Understanding how gut flora influences gut-brain communications has been the subject of significant research over the past decade. The broadening of the term “microbiota-gut-brain axis” from “gut-brain axis” underscores a bidirectional communication system between the gut and the brain. The microbiota-gut-brain axis involves metabolic, endocrine, neural, and immune pathways which are crucial for the maintenance of brain homeostasis. Alterations in the composition of gut microbiota are associated with multiple neuropsychiatric disorders. Although a causal relationship between gut dysbiosis and neural dysfunction remains elusive, emerging evidence indicates that gut dysbiosis may promote amyloid-beta aggregation, neuroinflammation, oxidative stress, and insulin resistance in the pathogenesis of Alzheimer’s disease (AD). Illustration of the mechanisms underlying the regulation by gut microbiota may pave the way for developing novel therapeutic strategies for AD. In this narrative review, we provide an overview of gut microbiota and their dysregulation in the pathogenesis of AD. Novel insights into the modification of gut microbiota composition as a preventive or therapeutic approach for AD are highlighted.

## Introduction

**Fig. 1:**
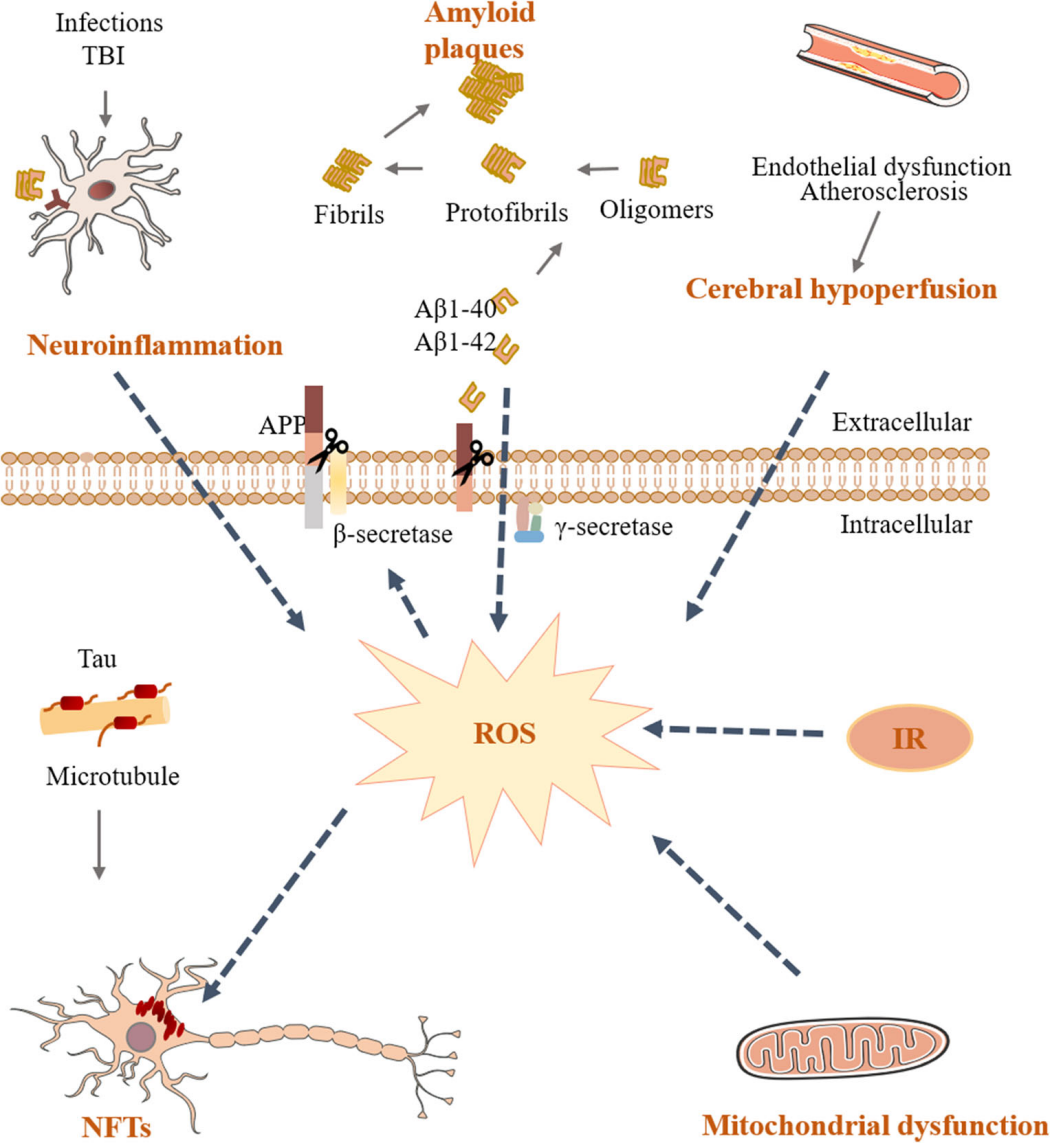
Key factors in the pathogenesis of AD. amyloid plaques and intracellular NFTs, neuroinflammation, mitochondrial dysfunction, OS, IR, and chronic cerebral hypoperfusion are the main causes of AD development. These factors are related to each other directly or indirectly. Cerebral hypoperfusion due to advanced atherosclerosis or endothelial dysfunction, IR, and mitochondrial dysfunction lead to an elevation in ROS levels which results in overexpression and increased processing of APP, hyperphosphorylation of tau, and NFT pathology leading to neuronal death. Aβ, TBI, and infections are some of the factors that can elicit inflammation. Abbreviations: *Aβ* amyloid-beta, *AD* Alzheimer’s disease, *APP* amyloid precursor protein, *IR* insulin resistance, *NFT* neurofibrillary tangle, *OS* oxidative stress, *ROS* reactive oxygen species, *TBI* traumatic brain injury

Alzheimer’s disease (AD) is a progressive neurodegenerative disorder of the central nervous system (CNS) characteristic of gradual cognitive decline [1]. The presence of extracellular amyloid-beta (Aβ) deposition as neuritic plaques and intracellular accumulation of hyperphosphorylated tau as neurofibrillary tangles (NFTs) remain the primary neuropathological criteria for AD diagnosis [1]. As the most prevalent form of dementia, AD has emerged as a global public health priority affecting an estimated total of 50 million people worldwide [2]. The consistently growing prevalence and the heavy burden of AD render it more urgent than ever for researchers to dissect the mechanisms underlying the pathogenesis of AD and to seek disease-modifying therapies (Fig. 1).

Since it was first proposed by Hardy and Higgins in 1992, the amyloid cascade hypothesis has been the dominant theory of AD pathogenesis which holds that the accumulation of Aβ peptides derived from amyloid precursor protein (APP) is the initial event of AD pathogenesis [3]. Over the ensuing decades, this hypothesis has evolved from a neuron-centric linear cascade, which postulates that Aβ results in hyperphosphorylated tau and neurodegeneration, to an integrative model that also involves feedback and feedforward responses of impaired vasculature, oxidative stress (OS), microgliosis, and dysregulation of neuronal proteolysis [4, 5]. Extensive research suggests that aggregated, hyperphosphorylated forms of tau may also lead to synaptic communication disturbance as well as neuronal death [6]. Although candidate drugs that affect the formation, aggregation, and clearance of Aβ and tau have yielded encouraging results in some preclinical trials, none have progressed to the clinical stage [2, 7].

Mounting evidence suggests that neuroinflammation is not just a consequence but a vital contributor to the development and progression of AD. As such, the “inflammation hypothesis” is emerging as a surrogate mechanism of AD (Fig. 1). In this scenario, synaptic impairment and neuronal death are at least partially mediated by excessive or non-resolving innate immune activation [8]. Extrinsic factors like infections and traumatic brain injury (TBI) can possibly interfere with the central immune homeostasis and accelerate disease progression [9, 10]. The “inflammation hypothesis” was fueled by the discovery of inflammation susceptibility genes (e.g., *CD33*, *TREM2*) for AD through large-scale genome-wide association studies [11, 12].

Extensive research argues mitochondrial dysfunction [13], insulin resistance (IR) [14], and cerebral hypoperfusion [15] may mediate, drive, or possibly even initiate pathologic molecular cascades in AD and finally promote Aβ accumulation, tau hyperphosphorylation, synaptic degeneration, and neuronal dysfunction. However, current hypotheses in this regard are unable to satisfactorily explain the etiology and underlying pathophysiological mechanisms of AD. In recent years, the microbiota-gut-brain axis has emerged as a focal point of biomedical research and a potential therapeutic target for the treatment of CNS disorders [16, 17]. In particular, dysfunction of the microbiota-gut-brain axis has been implicated in the pathogenesis of AD [19]. In this review, we will summarize the knowledge on the characteristics of the gut microbiota and the communication pathways of the microbiota-gut-brain axis, analyze the role of dysbiosis of the gut microbiota in the pathogenesis of AD, and highlight the modification of gut microbiota composition as a preventive or therapeutic approach for AD.

## Human Gut Microbiota

The human gut is an anaerobic bioreactor with a diverse population of microorganisms, including bacteria, yeast, archaea, viruses, protozoa, and parasites such as helminths, collectively known as microbiota, which occupy different niches of the mucosal surfaces in the gastrointestinal (GI) tract [16, 20]. The application of DNA sequencing and metagenomic and metabolomic analysis technologies has reshaped our view of human gut flora and provided new insights into the characterization of microbiota and its intricate interplay with human health [18]. The microorganisms residing in the gut make up the vast majority of the human microbial population, including at least 1000 different bacterial species and approximately 150 times as many genes as in the human genome [21, 22]. The distinct microenvironment of each gut compartment selects the growth of specific microbiota, with the distal gut being the predominant habitat of the gut microbial community [22]. *Firmicutes* and *Bacteroidetes* constitute the most abundant phyla in human intestinal microbiota [23]. Initial colonization of the GI tract by gut microbiota is thought to commence at birth when the infant becomes exposed to maternal microbiota and other environmental factors during birth [24]. Importantly, the gut microbiota of vaginally born infants closely resembles the microbial compositions of the mothers’ vagina, while newborns delivered via cesarean section are enriched with microbes found on human skin and in the surrounding environment [25]. Gut microbiota composition exhibits a large interindividual variability and heterogeneity that may be explained by an influence of both extrinsic, e.g., diet, antibiotics, lifestyle, and disease, and intrinsic factors, e.g., genetics [26, 27]. The diverse commensal microbiota undergoes dynamic changes throughout life, as is evidenced by the fact that the number of species and the richness of gut microbiota composition decline prominently with age [28]. Intestinal microbiota is proposed as an essential “organ” which imparts substantial physiological functions related to innate immunity, appetite, and energy metabolism locally and systematically [16, 29]. Abnormal changes in the composition of gut flora, a phenomenon known as dysbiosis, is also directly involved in the pathophysiology of diseases affecting several distant organs [30]. Gut dysbiosis may be associated with pathologies such as asthma [31], cardiovascular disease [32], type 2 diabetes mellitus (T2DM) [33], renal failure [34], and sarcopenia [35].

## The Microbiota-Gut-Brain Axis in Health and Disease

**Fig. 2:**
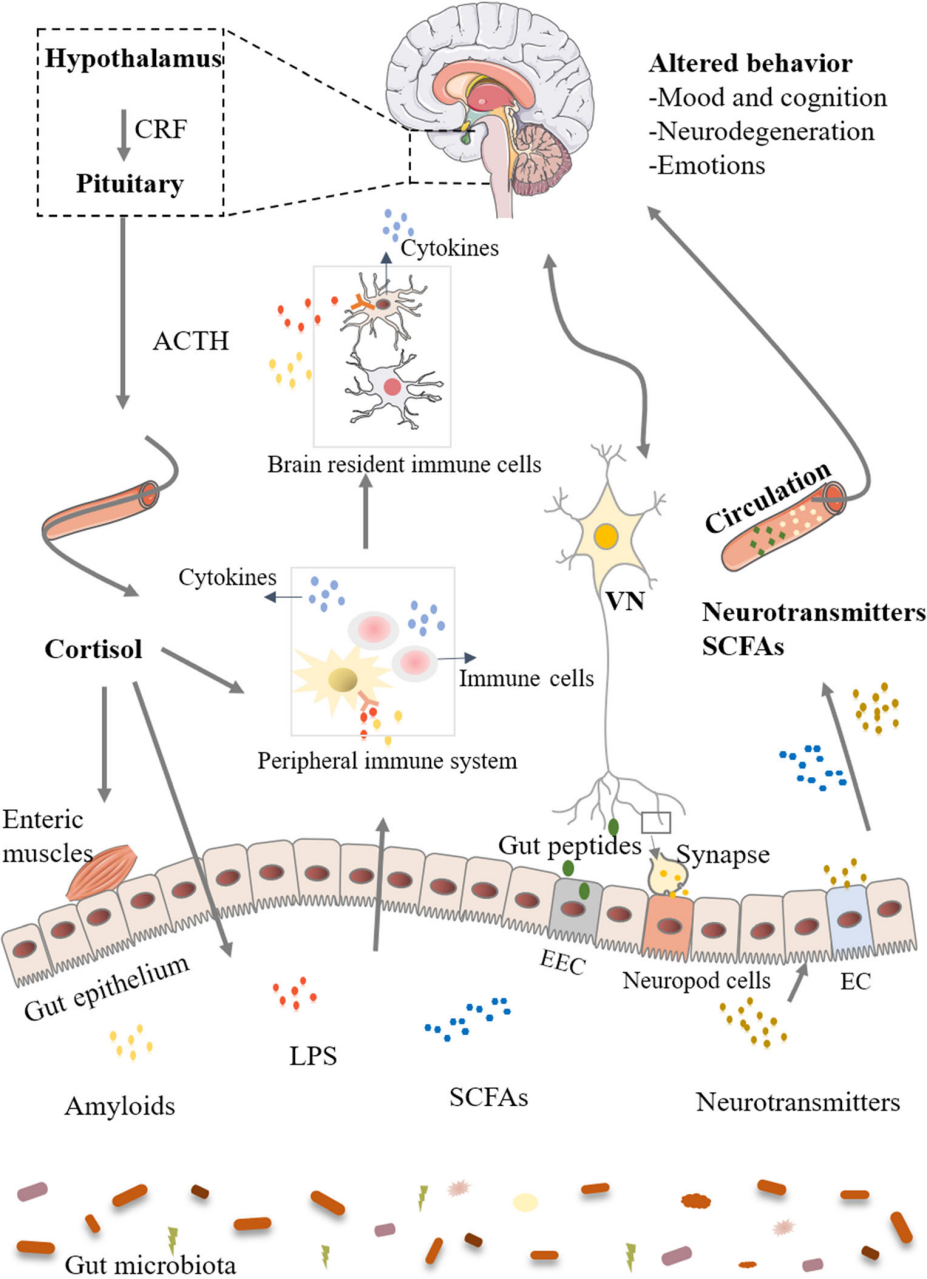
Communication between gut microbiota and the brain. Communication pathways between gut microbiota and the brain include metabolic, endocrine, neural, and immunological pathways which can work independently or cooperatively: (1) gut microbiota metabolites, including SCFAs, neurotransmitters, and amyloids, may reach the brain to regulate neurological function; (2) gut microbiota interacts with the HPA axis, regulating brain function and gut microbiota composition; (3) direct activation of the vagus nerve from the enteric nervous system is transmitted to the brain; (4) MAMPs such as LPS can activate both the peripheral and the central immune system. Abbreviations: *ACTH* adrenocorticotropic hormone, *CRH* corticotropin-releasing hormone, *EC* enterochromaffin cell, *EEC* enteroendocrine cell, *HPA* hypothalamus-pituitary-adrenal, *LPS* lipopolysaccharide, *MAMPs* microbial associated molecular patterns, *SCFAs* short-chain fatty acids, *VN* vagus nerve

The term “microbiota-gut-brain axis” broadened from “gut-brain axis” indicates the significant role of gut microbiota in modulating brain function. Albeit nascent in terms of the delineation of the mode of communication between gut microbiota and the brain, research using germ-free (GF) mice, antibiotic treatments, and prebiotic/probiotic complementation has provided persuasive evidence for several major potential pathways underlying the two-way communications between the GI tract and the CNS. At least five separate lines of evidence converged to support the hypothesis that gut microbiota can effectively communicate with the brain. First, it has long been known that a clinical situation, i.e., hepatic encephalopathy, is associated with gut dysbiosis and can be broadly treated by targeting the microbiota with antibiotics in humans; second, studies in GF animals showed that the brain function is affected by the absence of microbiota; third, low-level infections alter the behaviors of animals and humans even in the absence of immune activation; fourth, specific strains of exogenous bacteria alter the behaviors of animals and humans; and finally, antibiotic administration has long-lasting effects on the nervous system [16, 17]. In this section, we will summarize the updated knowledge on role of the microbiota-gut-brain axis in health and disease from the perspective of metabolites, endocrine regulation (the hypothalamic-pituitary-adrenal [HPA] axis), neural transmission, and immunomodulation (Fig. 2) [16, 17].

### Metabolites

The ability of bacteria to produce bioactive products provides a mechanistic basis to understand the role of gut microbiota in modulating central physiological and pathological processes [36]. Moreover, the emerging notion that the interplay between bacterial products and the brain can modulate behavior is intriguing. Different bacterial genera and species produce gamma-amino butyric acid (GABA), serotonin (5-HT), histamine, and dopamine (Table 1), which are all involved in a range of mood-related, behavioral, and cognitive functions as neurotransmitters or neurotransmitter precursors [43, 44]. During metabolism, the host and its gut microbiota coproduce a spectrum of metabolites, such as short-chain fatty acids (SCFAs), that are essential for host health [45]. SCFAs, which mainly consist of acetate, propionate, and butyrate, function through either G protein coupled receptors or histone deacetylases [46, 47]. Such microbial products are active mediators of gut-brain communication and may serve as potential therapeutic targets for neurodevelopmental and neurodegenerative disorders.

**Table 1:** Common species of gut microbiota and effects of their metabolites on the central nervous system

Gut microbiota	Metabolites	Effects on the central nervous system function	References
*Lactobacillus*, *Bifidobacterium*	Gamma-aminobutyric acid (GABA)	The predominant inhibitory neurotransmitter, regulates mood, behavioral and cognitive functions	[37]
*Bifidobacterium infantis*, *Streptococcus*, *Escherichia*, *Enterococcus*, *Lactococcus*, *Lactobacillus*, *Candida*	Serotonin (5-HT)	Neurotransmitters, regulate emotions	[38]
*Escherichia*, *Bacillus*, *Lactococcus*, *Lactobacillus*, *Streptococcus*	Dopamine	Regulate mental activities and motor functions, cognitive functions such as learning and memory	[38]
*Lactobacillus*, *Bacillus*	Acetylcholine	Cognitive, memory, social life ability, self-care ability, and emotional personality	[39]
*Bacteroides*, *Bifidobacterium*, *Propionibacterium*, *Eubacterium*, *Lactobacillus*, *Clostridium*, *Roseburia*, *Prevotella*	Short-chain fatty acids (SCFA)	Decrease permeability of the blood-brain barrier (BBB), promote the synthesis and secretion of neurotransmitters and hormones, reduce inflammation	[40]
*Lactococcus*, *Lactobacillus*, *Streptococcus*, *Enterococcus*	Histamine	Regulate sleep and cognition	[41, 42]

### Endocrine Regulation

The HPA axis, as one of the main neuroendocrine systems in the human body, is a principal regulator of the response to stress [48]. In a pioneering study linking gut microbiota to the HPA axis, plasma adrenocorticotropic hormone and/or corticosterone elevation in response to restraint stress was more remarkable in adult GF mice than in specific-pathogen-free (SPF) mice (with a normal composition of microbiota and no specific pathogens) [49]. The exaggerated stress response in GF mice could be partially reversed at 9 weeks of age by reconstitution with feces from the control mice [50]. Paradoxically, reduced anxiety-like behavior and central neurochemical change were observed in GF mice as compared to SPF mice [51]. Significant decrease in N-methyl-D-aspartate receptor subunit NR2B mRNA expression in the central amygdala and 5-hydroxytryptamine 1A receptor mRNA expression in the dentate gyrus may contribute to the altered HPA function in GF animals [51]. Although GF animals have been a cornerstone for investigating whether gut microbiota is involved in HPA axis regulation, they have many limitations in terms of dysregulated hormone signaling, aberrant neurodevelopment, and an impaired immune system due to a lack of exposure to microorganisms since birth [16]. Translational studies are therefore still limited, because no equivalent obliteration of gut microbiota can be conducted in humans.

### Neural Transmission

The vagus nerve (VN) originates in the medulla oblongata of the CNS and innervates numerous structures such as the heart and the GI tract, which is the key neural pathway between the gut and the brain, containing 80% and 20% of afferent and efferent fibers, respectively [52, 53]. As a vast variety of chemical and mechanosensitive receptors are expressed on vagal afferents, and due to their role in interoceptive awareness, they respond to a variety of mechanical, chemical, and hormonal stimuli from gut microbiota and transfer gut information to the CNS [54]. However, this chemo- and mechanosensitive perception cannot be conducted directly, because vagal afferents do not cross the epithelial layer [55]. Interestingly, a subtype of gut enteroendocrine cells (EECs) were found to synapse with vagal neurons and transmit the information to the brain directly; these EECs were named neuropod cells [56]. Perhaps the most striking observation regarding the role of the VN in the microbiota-gut-brain axis comes from vagotomy studies. Mice treated with *Lactobacillus rhamnosus* showed reduced anxiety- and depression-related behavior, which was not observed in mice with VN ablation [57]. Furthermore, vagotomy reduced the proliferation and survival of newborn cells and decreased the number of immature neurons and the activation of microglia in the dentate gyrus of the hippocampus [58]. Whether VN targeting by stimulation or vagotomy translates to microbial-based CNS therapeutics remains a tempting possibility and merits further investigation.

### Immunomodulation

The densest concentration of immune cells, such as B cells, T cells, macrophages, and dendritic cells, is found in the intestine. Gut microbiota can profoundly affect the development of organized lymphoid structures and the activation of both the innate and adaptive immune systems [59, 60]. Microbiota-host immune interactions in the gut lead to the release of proinflammatory mediators, e.g., cytokines and chemokines, and specific antibodies involved in the regulation of brain immunity. Metabolites produced by gut microbiota also regulate the maturation, differentiation, and activation of microglia and astrocytes, which mediate several neurophysiological processes, including maintenance of blood-brain-barrier (BBB) integrity, neural development, neurotransmission, and CNS immune activation [61, 62]. Therefore, a complex immunoregulatory network of interactions exists among gut microbiota, the intestinal mucosal immune system, and the brain.

## Roles of the Microbiota-Gut-Brain Axis in the Pathogenesis of AD

**Fig. 3:**
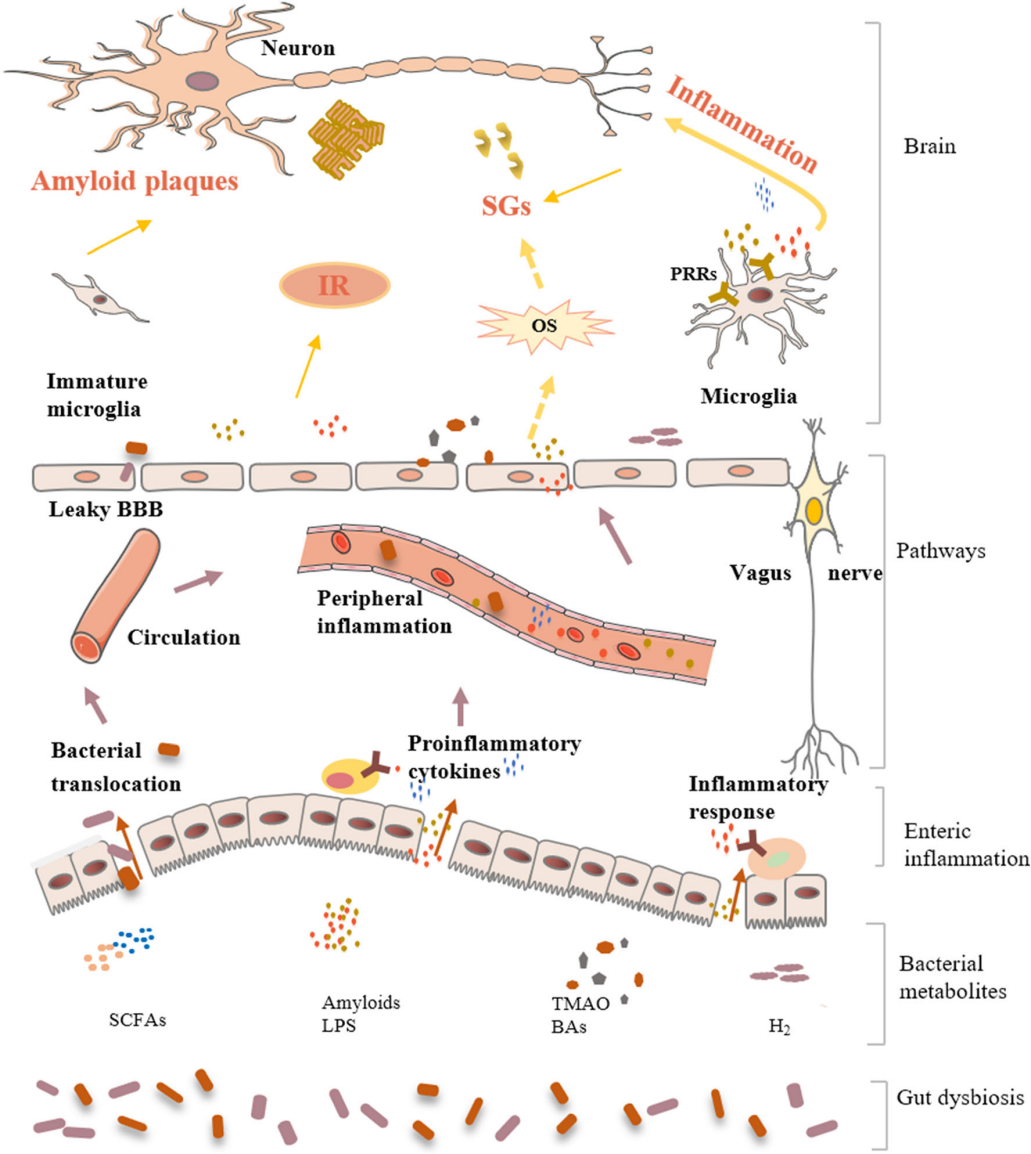
Impact of gut dysbiosis on AD. Gut dysbiosis induces the decrease of beneficial substances (such as SCFAs and H_2_) and the increase of harmful substances (such as amyloids and TMAO), which causes the intestinal mucosal barrier and BBB to become permeable, activates peripheral immune responses, and increases peripheral and central OS levels. Finally, gut dysbiosis contributes to AD pathology progression by increasing amyloid plaque formation, neuroinflammation, SGs, and IR. Arrows indicate the direction of the effect. Yellow arrows with dashed lines indicate that no studies have explored this putative relationship yet in the AD-gut microbiome field. Abbreviations: *AD* Alzheimer’s disease, *BAs* bile acids, *BBB* blood-brain barrier, *LPS* lipopolysaccharide, *H*_*2*_ hydrogen, *IR* insulin resistance, *OS* oxidative stress, *PRRs* pattern-recognition receptors, *SCFAs* short-chain fatty acids, *SGs* stress granules, *TMAO* trimethylamine N-oxide

Alterations of gut microbiota in animals and patients with AD are summarized in Table 2. Key questions as to how the axis contributes to the onset and/or progression of AD, however, remain unanswered. In this section, we review the roles of the microbiota-gut-brain axis in the pathogenesis of AD so as to elucidate the possible pathophysiological mechanism underlying the modulation of gut microbiota in AD (Fig. 3).

**Table 2:** Summary of studies concerning the alterations of the gut microbiota in AD

Experimental subject	Methods	Main findings	Reference
Patients with cognitive impairment and brain amyloidosis	Microbial DNA qPCR assay	*Escherichia*/*Shigella*↑, *E. rectale*↓	[63]
AD patients	16S rRNA sequencing	*Bacteroides*, *Actinobacteria*, *Ruminococcus*, *Lachnospiraceae*, and *Selenomonadales* at taxonomic levels	[64]
AD patients	16S rRNA sequencing	*Firmicutes* and *Bifidobacterium*↓, *Bacteroidetes*↑	[65]
AD patients	PCR	Significant difference in the gut microbial genotypes between the AD and control human populations	[66]
AD patients	16S rRNA sequencing	Bacterial population in the brain ↑	[67]
APP/PS1 transgenic mice	16S rRNA sequencing	The microbiota composition and diversity were perturbed	[68]
APP/PS1 transgenic mice	16S rRNA sequencing	*Proteobacteria* and *Erysipelotrichaceae* increased with age. Total *Bacteroidetes* remain stable. The inflammation-related family *Erysipelotrichaceae* was more abundant in aging	[69]
APP/PS1 transgenic mice	16S rRNA sequencing	Microbiota diversity was decreased *Odoribacter* and *Helicobacter*↑	[70]
Symptomatic Tg2576 mice	16S rRNA sequencing	The percentage abundance of *Firmicutes* and *Bacteroidetes* phyla was significantly higher. *Lactobacillusi* ↑	[71]

### Associations Between the Microbiota-Gut-Brain Axis and Aβ Accumulation

Neurological disorders such as AD, Parkinson’s disease (PD), and amyotrophic lateral sclerosis are characterized by the gradual accumulation of abnormal proteins in the CNS [72]. The proclamation “all disease begins in the gut,” purported by the Greek physician Hippocrates 2000 years ago, has been an intriguing one and continues to influence medical researchers and practitioners [16]. One hypothesis regarding the pathogenesis of PD claims that the initial induction and subsequent aggregation of α-synuclein probably originate in the enteric nervous system and spread upwards progressively to the CNS, propagating trans-synaptically from nerve cell to nerve cell in a virtually self-promoting pathological process [73]. Gut dysbiosis is mainly characterized by an increase in the *Firmicutes*/*Bacteroidetes* ratio, which could cause intestinal APP accumulation since the earliest stages of AD [74]. Changes in the composition of gut microbiota in APP/PS1 mice were related to an increase in Aβ levels in the CNS and impaired spatial learning and memory [70]. Moreover, aberrant accumulation of Aβ in myenteric neurons and activation of intestinal innate immunity appear before the onset of CNS neuroinflammation in AD mice [75]. Likewise, gut dysbiosis, intestinal epithelial barrier dysfunction, and vascular Aβ deposition in the gut occur before the onset of cerebral Aβ depositions in Tg2576 mice (a transgenic mouse model of AD) [71]. The presence of Aβ deposits is also noted in intestinal autopsies of patients with AD [71]. Hence, a hypothesis which assumed that Aβ accumulation in the gut precedes that in the brain came into being. However, research aimed at elucidating the relationship between gut dysbiosis, intestinal Aβ accumulation, and AD onset is lacking and a causal relationship between them has not been established. Early manipulation of gut physiology and microbiota as a means to possibly reverse the pathology of AD needs further investigation.

Another possible mechanism is related to the release of certain bacterial metabolites by gut microbiota. SCFAs demonstrate efficacy in interfering with protein-protein interactions that are indispensable for Aβ assemblies [76]. The microbial-derived metabolite trimethylamine N-oxide (TMAO) has been implicated in the pathogenesis of AD as well [77]. TMAO causes cognitive deterioration and pathological processes in AD by increasing β-secretase activity and thus aggravating Αβ accumulation [77]. Moreover, gut microbiota, through the generation of TMAO, contributes to platelet hyperreactivity by enhancing the stimulus-dependent release of calcium ions from intracellular stores, promoting the Aβ produced in platelets to enter the circulation and reach the brain [78–80]. Through a mechanism very similar to that of prion molecules, Aβ may seed in the brain from neuron to neuron, contributing to the progression of cytopathological lesions in AD [72]. These data suggest that specific personalized nutrition interventions might represent an effective strategy to modify the production and aggregation of Aβ.

Increased circulating bacterially produced bile acids (BAs) may increase BBB permeability via the disruption of tight junctions [81, 82] and permit BAs or peripheral cholesterol to reach the CNS [83]. Mounting cellular cholesterol in the brain leads to direct binding to APP and thus facilitates APP insertion into the phospholipid monolayers of the lipid rafts where Aβ formation takes place, eventually promoting the production of Aβ [84]. More importantly, cholesterol accumulation in the brain may be induced via effects mediated by BAs on the farnesoid X receptor, which downregulates the expression of the cholesterol-metabolizing enzyme CYP46A [85]. From this perspective, BAs perturb cholesterol elimination pathways, cause cholesterol accumulation, and further increase Aβ production. Of note is the contribution of bacterial amyloids to Aβ accumulation. A well-described bacterial amyloid is curli, which is produced by *Escherichia coli.* Through molecular mimicry, bacterial amyloids may act as prion proteins to cross-seed and aggregate host amyloids [86]. Other amyloids produced by microbes include FapC by *Pseudomonas fluorescens*, phenol-soluble modulins by *Staphylococcus aureus*, chaplins by *Streptomyces coelicolor*, and MccE492 by *Klebsiella pneumonia* [87].

Microglial activation is associated with AD pathology [88] and phagocytic microglia are crucial for the uptake and engulfment of soluble Aβ species and the phagocytosis of insoluble fibrillar Aβ deposits [89]. Activated microglia clustering around amyloid deposits may constitute a barrier that can compact amyloids, minimize damage to adjacent neuropils, and decrease the incorporation of new neurotoxic Aβ into existing plaques [90]. Notably, gut microbiota appears to be a prerequisite for microglia maturation and function under homeostatic conditions [61]. GF mice and SPF mice, both with reduced gut microbiota complexity, displayed defects in microglia characterized by altered cell proportions and immature phenotypes which could successfully be rescued by supplementation with SCFAs or by re-introducing live and complex microbiota, respectively [61]. Microglia from adult GF mice also exhibited downregulated expression of genes associated with microglial maturation [61]. Microglia in SPF mice lacking free fatty acid receptor 2 (a SCFA receptor) displayed a phenotype nearly identical to that observed in GF mice [61]. These findings suggest that microglia are highly sensitive to perturbations in the gut microbial community and metabolites.

Taken together, although the pathogenic role of Aβ in the pathogenesis of AD needs to be further clarified, gut dysbiosis may contribute to the neuronal damage in AD in an Aβ-dependent mechanism. Future research should focus on whether gut microbiota represents an important hinge between AD and its risk factors.

### Neuroinflammation and Microbiota-Mediated Alteration of BBB Permeability

The BBB is a multilayered unit, comprising specialized brain endothelial cells linked by tight protein junctions in the microvasculature, which acts as a semipermeable barrier to control the passage and exchange of molecules and nutrients between the circulatory system and the brain parenchyma [91]. Structural and functional disruption of the BBB in AD may be an early and important step in the pathogenesis [92]. A pioneering study reported that GF mice showed increased permeability of the BBB, which may partly be the consequence of disorganized tight junctions due to reduced expression of tight junction proteins, especially occludin and claudin-5; the reduced BBB permeability could be restored by recolonization of gut microbiota, implying a causal role for gut microbiota in ensuring the development of the BBB [93]. SCFAs can enter the circulation and decrease the permeability of the BBB by increasing the expression of endothelial tight junction proteins, especially occludin and claudin-5 [93, 94]. A propionate concentration as low as 1 μm can protect the BBB from OS by an NFE2L2-dependent mechanism [95]. These findings implicate the ability of gut microbiota to affect the permeability of the BBB. Impaired intestinal epithelial barrier integrity may permit unregulated translocation of pathogenic microbiota out of the gut, which then undergoes dissemination in the CNS via the impaired BBB [96]. Compared with those from control groups, brain samples from patients with AD showed an increase in bacterial populations [67]. Several studies have revived interest in a long-standing hypothesis that there may be a possible microbial origin for AD [97, 98]. One piece of seminal evidence is that Aβ exhibits characteristics of an anti-microbial peptide which is active against at least 12 different microorganisms [99]. Intriguingly, *Salmonella typhimurium* bacterial infection of the brains of 4-week-old 5xFAD mice led to the presence of Aβ in brain parenchyma, where it closely colocalized with the bacterial deposition [100]. Similarly, increased Aβ deposition was present in brain parenchyma sites containing deposits of herpes simplex virus type 1 [101]. However, it is ethically challenging for validation studies conducted on humans.

Gut microbiota also produces a remarkably complex array of proinflammatory neurotoxins that can cross the BBB and are pathogenic and highly detrimental to the homeostatic function of neurons in the CNS [16]. In particular, the role of lipopolysaccharide (LPS) generated by Gram-negative bacteria is relatively well documented. Bacterial LPS can bind with microglial cell receptors (TLR2, TLR4, and/or CD14) to initiate the production of a cascade of cytokines and chemokines via myeloid differentiation factor 88 (MyD88) and nuclear factor kappa beta (NF-κB)-dependent signaling pathways [102, 103]. Interestingly, the presence of LPS has been detected in the neocortex and hippocampus of patients with AD [104]. In a rodent model, TLR4 inhibited proliferation and neuronal differentiation upon LPS binding, while TLR2 enhanced hippocampal neurogenesis [105]. LPS appeared to efficiently activate NF-κB signaling to increase the levels of proinflammatory miRNA-146a and miRNA-155, which resulted in the downregulation of complement factor H expression and contributed to the onset of AD [106]. The inflammasome is a cytosolic macromolecular signaling platform which mediates activation of the cysteine protease caspase-1, leading to proteolytic processing and secretion of the proinflammatory cytokines interleukin (IL)-1β and IL-18 [107]. Inflammasome-mediated inflammation may represent a critical component of the inflammatory response in AD [108]. Extracellular LPS has also been reported to trigger microglial NOD-like receptor protein (NLRP)3 inflammasome activation [109]. Therefore, bacterial endotoxins may be internal contributors to inflammatory degeneration in the CNS. Aside from glial subsets, innate and adaptive immune cells, including perivascular macrophages, CD4^+^ T and CD8^+^ T cells, and mast cells, are also resident in the CNS [62].

Systemic inflammation is likely to interfere with the immunological processes of the brain and further promote AD progression. This hypothesis is supported by clinical studies of AD showing increased cognitive decline and exacerbation of neurodegeneration following acute and chronic systemic inflammation [110, 111]. Increase in levels of the proinflammatory bacteria *Escherichia*/*Shigella* and reduction in the levels of the anti-inflammatory bacteria *Eubacterium rectale* are associated with increased levels of IL-1β, CXCL2, and NLRP3 in the plasma of patients with cognitive impairment and brain amyloidosis [63]. The intestinal mucosal lymphoid tissue is regarded as the largest and most important human immune organ, containing 70–80% of the immune system of the whole body [112]. Microbe-associated molecular patterns (MAMPs) such as LPS can bind with various pattern-recognition receptors expressed on macrophages and dendritic cells in the gut, resulting in the production of inflammatory mediators such as proinflammatory cytokines [113]. A more permeable intestinal epithelial barrier permits these inflammatory mediators to enter the circulatory system and cause systemic inflammation [96]. Microbe-derived metabolites are additional contributors to systemic inflammation; for example, alterations of gut microbiota could lead to elevation of phenylalanine and isoleucine concentrations in the periphery of AD mice, which could provoke the infiltration of various immune cells, including T cells, B cells, natural killer cells, neutrophils, dendritic cells, and monocytes; CD4^+^ T helper (Th)1 cell levels were significantly correlated with M1 microglial activation in the brain during the progression of AD [114].

TBI is an important risk factor for developing AD later in life, though the mechanism behind this correlation is still unclear [10]. Gut microbiota may serve as a potential hub linking TBI, inflammation, and AD. Significant changes in gut microbiota composition at the genus and species level in injured mice highlighted the high probability of gut dysbiosis after TBI [115]. The systemic inflammatory response resulting from gut dysbiosis exerts an influence on the vulnerable and primed microglia following brain injury to further exacerbate neuroinflammation, which in turn predisposes or accelerates the onset and progression AD [116]. Similarly, gut microbiota may also be involved in a potential nexus between chronic psychological stress, inflammation, and AD. The HPA axis plays a significant role in the host’s response and adaptation to stress. Observations of elevated basal cortisol levels in patients with AD prompted the hypothesis that stress may predispose the brain to neurodegeneration [117]. Indeed, chronic psychological stress leads to microglial activation, characterized by an exaggerated release of proinflammatory cytokines and chemokines [118]. Physiological stress may alter the integrity of the intestinal epithelial barrier and the composition of gut microbiota in association with release of proinflammatory cytokines, such as IL-1β [96, 119], that could activate the HPA axis; in this manner, gut microbiota can dramatically modify the body’s responsiveness to stress [49, 50]. In this perspective, gut microbiota located at the key position in the stress-inflammation loop might be actively involved in neuroinflammation and AD pathogenesis.

### Does Gut Microbiota Modulate OS?

Non-polysomal RNA and mRNA-binding proteins (RBPs) are the major components of stress granules (SGs), which form through a process of liquid-liquid phase separation (LLPS) when exposed to multiple types of stresses [120]. SGs are generally dynamic structures that rapidly form and disassemble with acute stress, while persistent SGs form as a consequence of chronic environmental stress and have been implicated in the pathogenesis of AD [121].

SGs may even be the breeding ground for the aberrant aggregation of pathological tau [121, 122]. Interactions between tau and SGs stimulate the formation of tau aggregates [123]. Tau undergoes LLPS with RNA and forms tau droplets which turn into aggregates to create the initial site of filament assembly, a process associated with AD pathogenesis [124]. SGs have a high concentration of mRNA, which probably enhances the conversion of tau into droplets [122]. Importantly, tau co-localizes strongly with RBPs and does not undergo LLPS in the absence of RBPs [121]. These findings advance our understanding of how these processes trigger tau protein aggregation and accelerate AD pathophysiology.

OS represents a condition of imbalance between the accumulation and elimination of ROS, which has various deleterious effects on the body [125]. OS is a well-recognized condition that facilitates SG formation [126]. Gut microbiota may influence the levels of OS in the CNS either by increasing the oxidant components or by interfering with anti-oxidant systems [127]. Gut microbiota promotes ROS generation within the gut epithelia [128], which in turn disturbs gut microbiota composition and functionality and makes the intestinal epithelial barrier more permeable [129, 130]. Neurotoxic substances such as LPS may reach the CNS via the VN or systemic circulation, promoting microglial activation and neuroinflammation, which produces even more ROS [131]. Another mechanism is mostly speculative but is pertinent to the hypothesis that the oxidative state of the CNS could be regulated by gut microbiota via the production of various metabolites. Decreased butyrate levels could impair mitochondrial functions, resulting in significant ROS production in Crohn’s disease [132]. Similarly, we speculate that the relative abundance of butyrate producers may cause mitochondrial dysfunction in the brain and increased production of ROS. Hydrogen (H_2_), a highly diffusible bioactive gas with anti-oxidant properties, was found to be produced mainly by strains of the genus *Clostridium*, anaerobic cocci, and members of the *Enterobacteriaceae* family [133]. Gut dysbiosis may result in low H_2_ production in the gut, and less gas is subsequently transferred to the portal vein, limiting the availability of the gas in the CNS [133]. Alterations of gut microbiota may in this regard interfere with the intracranial OS level and further favor SG formation. SGs have progressively gained recognition as essential contributors to the pathogenesis of AD [121], which provides a new dimension to the understanding of gut microbiota and AD pathogenesis.

### Does Gut Microbiota Affect AD Progression Via IR?

Compelling preclinical and clinical evidence supports the hypothesis that impaired insulin signaling may be associated with AD pathogenesis [134]. Considerable overlap has been identified in the molecular, biological, pathophysiological, and metabolic dysfunctions in AD and T2DM. IR is the disturbance of glucose regulation characterized by higher insulin levels. As a consequence of tissue desensitization to the action of insulin, IR occurs in peripheral tissues in patients with diabetes and obesity and has recently been shown to develop in AD brains [135]. In light of these observations, AD has also been termed as “type 3 diabetes,” which is viewed in a sense as a degenerative metabolic disease [136]. However, insulin signaling impairment has been observed even in the brains of patients with AD who are not diabetic [137]. Whether IR in AD and T2DM is a parallel phenomenon arising from coincidental roots in aging or whether there is an underlying mechanistic link is unclear.

Colonization of GF mice with gut microbial communities harvested from *ob*/*ob* mice resulted in increased body fat associated with IR [138]. Moreover, interventions involving probiotics and antibiotics improved insulin signaling and strengthened glucose control [139, 140]. Alterations in gut microbiota can modulate behaviors through the regulation of brain insulin sensitivity. Increased brain insulin signaling sensitivity and reduced signs of anxiety and depression were observed in mice with diet-induced obesity treated with oral metronidazole or vancomycin [141]. Furthermore, manipulation of gut microbiota through oral administration of probiotics ameliorated impaired glucose metabolism and cognitive dysfunction in a mouse model of AD [142]. The current data therefore allow us to hypothesize that changes in gut microbiota may contribute to IR in patients with AD. Future research efforts should be dedicated to better clarify the causal relationship between gut microbiota, IR, and AD. The phenomenon of IR is essential to our understanding of the overlap between AD and T2DM. Given the cause-effect relationship between gut microbiota and IR, T2DM and AD may be parallel diseases arising from the same fundamental aging-related alterations in gut microbiota.

## Potential Therapeutic Strategies for AD Targeting the Microbiota-Gut-Brain Axis

### Diet

Diet represents a pivotal determinant of gut bacterial assembly and genetic composition [143]. Specific foods and dietary patterns can influence the composition and abundance of different types of bacteria in the gut, which can in turn maintain host homeostasis. The Mediterranean diet (MD), which includes high-level consumption of fruits, vegetables, legumes, and cereals, has long been described as a healthy dietary pattern [16, 17]. Higher MD slowed the progression of AD and conferred protection of 1.5 to 3.5 years against AD [144]. The MD confers anti-inflammatory effects which are often linked with an increase in *Bacteroides* and *Clostridium* phyla and decrease in *Proteobacteria* and *Bacillaceae* phyla [145]. Human intervention studies suggest that high-level consumption of plant-based food consistent with the MD can modulate the gut microbiome composition, increase fecal SCFA levels, and decrease urinary TMAO levels [146]. Microbiota-accessible carbohydrate supplementation could ameliorate Western diet-induced gut dysbiosis, intestinal epithelial barrier impairment, and systemic inflammation in mice [147]. Furthermore, microbiota-accessible carbohydrate supplementation reduced neuroglial activation and synaptic impairment, which were eliminated with the depletion of gut microbiota using wide spectrum antibiotics [147]. The MD may serve as a potential therapeutic intervention in the treatment of AD by modulating gut microbiota. Such healthy diets merit investigation for their potential benefits with regard to brain disorders. However, much more work is still needed to determine how diet and its components imbue their effects on the microbiota-gut-brain axis and to delineate whether the effects of diets on microbiota drive changes in overall brain function.

### Pharmacological Strategies

Disease-modifying therapies are still lacking for AD [148]. One of the latest intriguing breakthroughs is GV-971, a sodium oligomannate extracted from algae that has been demonstrated to result in robust and consistent cognitive improvements in a phase 3 clinical trial [114]. GV-971 was found to significantly decrease microglial activation and multiple brain proinflammatory cytokine levels by altering the composition of gut microbiota and reducing the peripheral concentrations of phenylalanine and isoleucine generated by gut microbiota [114]. The underlying mechanism of GV-971 that improves cognition via alleviation of neuroinflammation inspires us to attempt to reduce peripheral phenylalanine and isoleucine concentrations directly by diet or pharmacological strategies instead of regulating gut microbiota.

Neurotransmitters including acetylcholine, GABA, histamine, and serotonin, which can be produced by gut microbiota, are critical modulators to regulate the gut-brain axis and play a crucial role in AD pathogenesis [149]. Exogenous manipulation of the serum concentrations of these neurotransmitters by targeting the appropriate receptors or blocking their breakdown may benefit decreased symptomology and/or disease progression. The United States Food and Drug Administration has approved four acetylcholinesterase inhibitors (AChEIs), tacrine, donepezil, rivastigmine, and galantamine, for “disease-modifying” effects on AD [150]. Several different clinical trials on novel AChEIs and acetylcholine stimulation in AD are ongoing, with the purpose of evaluating the efficacy, safety, tolerability, and pharmacokinetics of these drugs [151]. Ongoing studies are also being conducted to test the possibility of using GABA modulators and GABA agonists as AD therapeutics [151]. H3 receptor antagonists/inverse agonist molecules could inhibit histamine release and reverse partial cognitive function loss in animal models [152]. Various serotonin-mimetic compounds, selective serotonin reuptake inhibitors, and 5-HT receptor agonists or antagonists were proven to be safe and also improved cognitive disturbances. So far, no metabolite-based therapies associated with neurotransmitters are available to prevent disease processes and/or relieve the cognitive functions in patients with AD. Given the complex and significant role of neurotransmitters in AD pathogenesis, research exploring the therapeutic potential of neurotransmitters AD cannot be ignored. However, multiple cohort and longitudinal studies are still needed to this end.

### Probiotics

The term “probiotics” was first coined in 1974 and has conceptually evolved to its current definition as “live microorganisms that modify microbiota toward a beneficial state” [153]. Beneficial effects of probiotic supplementation include induction of immunomodulation, protection against physiological stress, pathogen antagonism, and improvement of the intestinal epithelial barrier function [154]. Mice treated with probiotics showed increased spatial memory and significantly lower quantities of plaques in the hippocampus [155]. Probiotic supplementation could also considerably improve synaptic plasticity and significantly restore long-term potentiation in the Aβ-administered animals [156]. With long-term oral ProBiotic-4 (a complex probiotic preparation) administration, senescence-accelerated mouse prone 8 mice showed significant improvement in microbiota composition of the feces and brain, cerebral neuronal and synaptic injuries, and immune response activation [157]. The underlying mechanism is related to inhibition of both TLR4- and retinoic-acid-inducible gene-I-mediated NF-κB signaling pathways in the brain [157]. At present, only one clinical trial has been conducted to assess the effects of probiotic supplementation on AD [158]. Patients with AD provided with probiotics showed improvement in cognitive function and favorable changes in related plasma biomarkers such as malondialdehyde and serum triglyceride [158]. These findings suggest that probiotic supplementation may have the therapeutic potentials to block or reverse the progression of AD. However, clinical evidence is insufficient to reach conclusions regarding the recommendation of probiotics for patients with AD. The exact mechanism by which probiotics display effects in AD remains unclear. Probiotic supplementation in humans did not seem to change the composition of intestinal flora (at least based on 16s rRNA sequencing) but induced the effect of probiotics on behavior by temporarily changing the transcriptional state of the collective microbiome, which was later confirmed in GF mice and monozygotic twins [159].

Metatranscriptomic and metabolomic technologies are needed to assess the effects of probiotic intervention on gut microbiota in the host. Although probiotics have been widely promoted among the general public, many probiotic strains and formulations have contradictory clinical results [154]. More attention should be paid to the adverse effects of probiotics, which include systemic infection, GI side effects, gene transfer from probiotics to normal microbiota, harmful metabolic effects of probiotics, immune system stimulation, etc. [160]. Besides, future probiotic therapy for AD requires the development of means to tackle colonization resistance [154].

### Prebiotics

The current definition of prebiotics is “a substrate that is selectively utilized by host micro-organisms and confers a health benefit,” proposed by the International Scientific Association for Probiotics and Prebiotics [153]. Several different kinds of food ingredients are considered prebiotics, among which resistant starch (RS), insulin, fructooligosaccharides (FOS), galactooligosaccharides, and xylooligosaccharides are most frequently highlighted [161]. RS is known to exert a powerful influence on metabolic and systemic health and has been extensively studied in clinical trials and animal models for evaluating treatment potential [162]. RS2 has been shown to alter the abundance of at least some intestinal bacterial genera and species, including enrichment of *Ruminococcus bromii*, *Bifidobacterium adolescentis*, *Faecalibacterium prausnitzii*, and *E. rectale* and reductions in *Oscillospira*, *Lachnospiraceae*, and *Blautia* [163]. FOS are found in natural fruits and vegetables and can promote the growth of beneficial gut microbiota such as *Bifidobacterium* and *Lactobacillus* [164, 165]. FOS are effective in maintaining the diversity and stability of the microbial community, alleviating neuronal apoptosis and the swelling of brain tissues, regulating the synthesis and secretion of neurotransmitters, and downregulating the expression of tau and Aβ1-42 in the brain of rats with AD-like symptoms [166]. These findings suggest that the therapeutic effect of FOS on AD is at least partially mediated by targeting of the microbiota-gut-brain axis. Furthermore, FOS exerted beneficial effects against AD via regulating the gut microbiota glucagon-like peptide-1 (GLP-1)/GLP-1 receptor pathway in APP/PS1 transgenic mice [167]. In humans, prebiotic supplementation exerts relatively modest effects on the microbiota and alters the expression level of cytokine genes, which may be beneficial for the elderly [168]. Although nurturing beneficial gut microbiota using prebiotics may provide major benefits, basic knowledge on the delicate interactions between the host, gut microbiota, and prebiotics is still lacking. Many technical and pragmatic difficulties, such as ensuring it reaches the location where it can exert its potential therapeutic effect, remain to be solved. Randomized controlled studies with larger cohorts are needed to evaluate the effect of prebiotics in patients with AD and to provide clinical evidence that can be translated into clinical practice.

### Fecal Microbial Transplantation

Fecal microbial transplantation (FMT) is the transfer of prescreened donor stool into the GI tract of patients with the aim of increasing overall diversity and restoring the function of gut microbiota [169, 170]. At present, FMT is only recommended as a treatment for recurrent *Clostridium difficile* infection, although trials on human diseases ranging from inflammatory bowel disease to metabolic diseases, neurodegenerative diseases, and cancer are ongoing worldwide. FMT could improve cognitive deficits and lessen Aβ deposition in the brain of AD animals [171]. Frequent transfer and transplantation of fecal microbiota from WT mice to ADLP^APT^ mice reduced amyloid plaque and NFT formation, glial responses, and cognitive impairment; FMT reversed the abnormal expression of intestinal macrophage activity-related genes and the increase of circulating blood inflammatory monocytes in ADLP^APT^ recipient mice [19]. Given that fecal material is expected to be sourced perfectly from a highly organized stool bank and various routes of administration such as capsule, enema, or colonoscopy [172], opportunities to exploit FMT to treat AD are materializing and FMT may be significantly convenient and efficacious. However, due to the inherent limitations of rodent models of human brain disorders, it is advisable to caution against premature extrapolation from preclinical data. FMT clearly poses significant unique and complex challenges for both clinicians and regulators, including its poorly defined mechanisms of action, stool availability, donor selection, adverse effects, and the relative lack of long-term follow-up data. Technical standardization, safety assessment, stool bank services and management, and other aspects are still in their infancy and need to be further studied. In this context, the FMT methodology is gaining considerable attention in both preclinical and clinical research and is likely to develop rapidly over the next decade.

## Conclusions and Perspectives

Gut microbiota can modulate important processes, including microglia maturation and activation, neurogenesis, myelination, synaptic pruning, and BBB permeability. The microbiota-gut-brain axis links gut microbiota and the brain via metabolic, endocrine, neural, and immune pathways that are crucial for the maintenance of brain homeostasis. Emerging evidence indicates that gut dysbiosis may aggravate Aβ aggregation and neuroinflammation in the development of AD. Restoring and remodeling gut microbiota composition may result in a strategic breakthrough in the treatment and, more importantly, the prevention of AD.

The development of cheaper and faster sequencing and other biological techniques has provided new insights into the characterization of gut microbiota. Human studies exploring biocommunication pathways between microbiota and the brain over time have flourished thereafter. However, some key issues need to be addressed in the future. First, the definition of normal or healthy microbiome may be one of the biggest challenges. A better description of microbial community dynamics and metabolism will help demarcate “normal” and “abnormal” human gut microbiota and identify therapeutic targets for AD. Second, factors like diet, drugs, and health status may confound the research on gut microbiota and AD. Cross-sectional clinical studies have demonstrated specific changes in the composition and functionality of gut microbiota in patients with AD. Longitudinal studies combining metagenomics sequencing and in-depth phylogenetic analysis with a comprehensive phenotypic characterization of patients with AD using up-to-date omics (metagenomics, metabolomics, transcriptomics, and metatranscriptomics) are urgently needed. Third, instead of purely observational studies, causal and functional ones should be strengthened. Although association analysis may provide important information for cause-effect deduction, correlation does not necessarily mean causation. Fourth, basic research targeting the microbiota-gut-brain axis in AD merits optimization; specifically, appropriate model systems are to be carefully selected. Host-specific interactions with microbiota, differences in environment and exposure, and the structural complexity of the brain should be taken into consideration. Finally, translation of basic research results into clinically relevant effects in humans should be expedited. The majority of data on the role of gut microbiota in AD are based on animal studies. Preclinical animal studies frequently end up with unexpected failures during clinical transition due to unidentified reasons. Alternatively, future therapeutic interventions will likely be based on individual gut microbiota composition due to significant differences in gut microbiota configurations and compositions among human populations.

## References

[1] Long JM, Holtzman DM (2019). Alzheimer disease: an update on pathobiology and treatment strategies. Cell.

[2] Hodson R (2018). Alzheimer’s disease. Nature.

[3] Hardy JA, Higgins GA (1992). Alzheimer’s disease: the amyloid cascade hypothesis. Science.

[4] Selkoe DJ, Hardy J (2016). The amyloid hypothesis of Alzheimer’s disease at 25 years. EMBO Mol med.

[5] De Strooper B, Karran E (2016). The cellular phase of Alzheimer’s disease. Cell.

[6] Tapia-Rojas C, Cabezas-Opazo F, Deaton CA, Vergara EH, GVW J, Quintanilla RA (2019). It’s all about tau. Prog Neurobiol.

[7] Panza F, Lozupone M, Logroscino G, Imbimbo BP (2019). A critical appraisal of amyloid-β-targeting therapies for Alzheimer disease. Nat Rev Neurol.

[8] Heneka MT, Carson MJ, El Khoury J (2015). Neuroinflammation in Alzheimer’s disease. Lancet Neurol.

[9] Ashraf GM, Tarasov VV, Makhmutovа A, Chubarev VN, Avila-Rodriguez M, Bachurin SO, Aliev G (2019). The possibility of an infectious etiology of Alzheimer disease. Mol Neurobiol.

[10] Becker RE, Kapogiannis D, Greig NH (2018). Does traumatic brain injury hold the key to the Alzheimer’s disease puzzle?. Alzheimers Dement.

[11] Guerreiro R, Wojtas A, Bras J, Carrasquillo M, Rogaeva E, Majounie E, Cruchaga C, Sassi C, Kauwe JS, Younkin S, Hazrati L, Collinge J, Pocock J, Lashley T, Williams J, Lambert JC, Amouyel P, Goate A, Rademakers R, Morgan K, Powell J, St George-Hyslop P, Singleton A, Hardy J, Alzheimer Genetic Analysis Group (2013). TREM2 variants in Alzheimer’s disease. N Engl J Med.

[12] Naj AC, Jun G, Beecham GW, Wang LS, Vardarajan BN, Buros J, Gallins PJ, Buxbaum JD, Jarvik GP, Crane PK, Larson EB, Bird TD, Boeve BF, Graff-Radford NR, de Jager PL, Evans D, Schneider JA, Carrasquillo MM, Ertekin-Taner N, Younkin SG, Cruchaga C, Kauwe JSK, Nowotny P, Kramer P, Hardy J, Huentelman MJ, Myers AJ, Barmada MM, Demirci FY, Baldwin CT, Green RC, Rogaeva E, George-Hyslop PS, Arnold SE, Barber R, Beach T, Bigio EH, Bowen JD, Boxer A, Burke JR, Cairns NJ, Carlson CS, Carney RM, Carroll SL, Chui HC, Clark DG, Corneveaux J, Cotman CW, Cummings JL, DeCarli C, DeKosky ST, Diaz-Arrastia R, Dick M, Dickson DW, Ellis WG, Faber KM, Fallon KB, Farlow MR, Ferris S, Frosch MP, Galasko DR, Ganguli M, Gearing M, Geschwind DH, Ghetti B, Gilbert JR, Gilman S, Giordani B, Glass JD, Growdon JH, Hamilton RL, Harrell LE, Head E, Honig LS, Hulette CM, Hyman BT, Jicha GA, Jin LW, Johnson N, Karlawish J, Karydas A, Kaye JA, Kim R, Koo EH, Kowall NW, Lah JJ, Levey AI, Lieberman AP, Lopez OL, Mack WJ, Marson DC, Martiniuk F, Mash DC, Masliah E, McCormick WC, McCurry SM, McDavid AN, McKee AC, Mesulam M, Miller BL, Miller CA, Miller JW, Parisi JE, Perl DP, Peskind E, Petersen RC, Poon WW, Quinn JF, Rajbhandary RA, Raskind M, Reisberg B, Ringman JM, Roberson ED, Rosenberg RN, Sano M, Schneider LS, Seeley W, Shelanski ML, Slifer MA, Smith CD, Sonnen JA, Spina S, Stern RA, Tanzi RE, Trojanowski JQ, Troncoso JC, van Deerlin VM, Vinters HV, Vonsattel JP, Weintraub S, Welsh-Bohmer KA, Williamson J, Woltjer RL, Cantwell LB, Dombroski BA, Beekly D, Lunetta KL, Martin ER, Kamboh MI, Saykin AJ, Reiman EM, Bennett DA, Morris JC, Montine TJ, Goate AM, Blacker D, Tsuang DW, Hakonarson H, Kukull WA, Foroud TM, Haines JL, Mayeux R, Pericak-Vance MA, Farrer LA, Schellenberg GD (2011). Common variants at MS4A4/MS4A6E, CD2AP, CD33 and EPHA1 are associated with late-onset Alzheimer’s disease. Nat Genet.

[13] Swerdlow RH (2018). Mitochondria and mitochondrial cascades in Alzheimer’s disease. J Alzheimers Dis.

[14] Nguyen TT, Ta QTH, Nguyen TTD, Le TT, Vo VG (2020) Role of Insulin Resistance in the Alzheimer's Disease Progression. Neurochem Res 45(7):1481–1491 10.1007/s11064-020-03031-0 32314178

[15] Zhao Y, Gong CX (2015) From chronic cerebral hypoperfusion to alzheimer-like brain pathology and neurodegeneration. Cell Mol Neurobiol 35(1):101–110 10.1007/s10571-014-0127-9 PMC11486181 25352419

[16] Cryan JF, O'Riordan KJ, Cowan CSM, Sandhu KV, Bastiaanssen TFS, Boehme M, Codagnone MG, Cussotto S, Fulling C, Golubeva AV, Guzzetta KE, Jaggar M, Long- Smith CM, Lyte JM, Martin JA, Molinero-Perez A, Moloney G, Morelli E, Morillas E, O'Connor R, Cruz-Pereira JS, Peterson VL, Rea K, Ritz NL, Sherwin E, Spichak S, Teichman EM, Wouw MVD, Ventura-Silva AP, Wallace-Fitzsimons SE, Hyland N, Clarke G, Dinan TG (2019) The microbiota-gut-brain axis. Physiol Rev 99(4):1877–2013 10.1152/physrev.00018.2018 31460832

[17] Long-Smith C, O'Riordan KJ, Clarke G, Stanton C, Dinan TG, Cryan JF (2020) Microbiota-gut-brain axis: New therapeutic opportunities. Annu Rev Pharmacol Toxicol 60(1):477–502 10.1146/annurev-pharmtox-010919-023628 31506009

[18] Knight R, Vrbanac A, Taylor BC, Aksenov A, Callewaert C, Debelius J, Gonzalez A, Kosciolek T, McCall L, McDonald D, Melnik AV, Morton JT, Navas J, Quinn RA, Sanders JG, Swafford AD, Thompson LR, Tripathi A, Xu ZZ, Zaneveld JR, Zhu Caporaso QJG, Dorrestein PC (2018) Best practices for analysing microbiomes. Nat. Rev. Microbiol 16(7):410–422 10.1038/s41579-018-0029-9 29795328

[19] Kim MS, Kim Y, Choi H, Kim W, Park S, Lee D, Kim DK, Kim HJ, Choi H, Hyun DW, Lee JY, Choi EY, Lee DS, Bae JW, Mook-Jung I (2020). Transfer of a healthy microbiota reduces amyloid and tau pathology in an Alzheimer’s disease animal model. Gut.

[20] Sonnenburg JL, Sonnenburg ED (2019) Vulnerability of the industrialized microbiota. Science 366. 10.1126/science.aaw9255 10.1126/science.aaw9255 31649168

[21] Qin J, Li R, Raes J (2010). A human gut microbial gene catalogue established by metagenomic sequencing. Nature.

[22] Gill SR, Pop M, Deboy RT (2006). Metagenomic analysis of the human distal gut microbiome. Science.

[23] Eckburg PB, Bik EM, Bernstein CN, Purdom E, Dethlefsen L, Sargent M, Gill SR, Nelson KE, Relman DA (2005). Diversity of the human intestinal microbial flora. Science.

[24] Perez-Muñoz ME, Arrieta MC, Ramer-Tait AE, Walter J (2017). A critical assessment of the “sterile womb” and “in utero colonization” hypotheses: implications for research on the pioneer infant microbiome. Microbiome.

[25] Dominguez-Bello MG, Costello EK, Contreras M, Magris M, Hidalgo G, Fierer N, Knight R (2010). Delivery mode shapes the acquisition and structure of the initial microbiota across multiple body habitats in newborns. Proc Natl Acad Sci U S A.

[26] Falony G, Joossens M, Vieira-Silva S, Wang J, Darzi Y, Faust K, Kurilshikov A, Bonder MJ, Valles-Colomer M, Vandeputte D, Tito RY, Chaffron S, Rymenans L, Verspecht C, de Sutter L, Lima-Mendez G, D’hoe K, Jonckheere K, Homola D, Garcia R, Tigchelaar EF, Eeckhaudt L, Fu J, Henckaerts L, Zhernakova A, Wijmenga C, Raes J (2016). Population-level analysis of gut microbiome variation. Science.

[27] Schmidt TSB, Raes J, Bork P (2018). The human gut microbiome: from association to modulation. Cell.

[28] Claesson MJ, Cusack S, O'Sullivan O, Greene-Diniz R, de Weerd H, Flannery E, Marchesi JR, Falush D, Dinan T, Fitzgerald G, Stanton C, van Sinderen D, O'Connor M, Harnedy N, O'Connor K, Henry C, O'Mahony D, Fitzgerald AP, Shanahan F, Twomey C, Hill C, Ross RP, O'Toole PW (2011). Composition, variability, and temporal stability of the intestinal microbiota of the elderly. Proc Natl Acad Sci U S A.

[29] Valdes AM, Walter J, Segal E, Spector TD (2018). Role of the gut microbiota in nutrition and health. BMJ.

[30] Schirmer M, Garner A, Vlamakis H, Xavier RJ (2019). Microbial genes and pathways in inflammatory bowel disease. Nat Rev Microbiol.

[31] Durack J, Kimes NE, Lin DL, Rauch M, McKean M, McCauley K, Panzer AR, Mar JS, Cabana MD, Lynch SV (2018). Delayed gut microbiota development in high-risk for asthma infants is temporarily modifiable by Lactobacillus supplementation. Nat Commun.

[32] Tang WHW, Bäckhed F, Landmesser U, Hazen SL (2019). Intestinal microbiota in cardiovascular health and disease: JACC state-of-the-art review. J Am Coll Cardiol.

[33] Zhao L, Zhang F, Ding X, Wu G, Lam YY, Wang X, Fu H, Xue X, Lu C, Ma J, Yu L, Xu C, Ren Z, Xu Y, Xu S, Shen H, Zhu X, Shi Y, Shen Q, Dong W, Liu R, Ling Y, Zeng Y, Wang X, Zhang Q, Wang J, Wang L, Wu Y, Zeng B, Wei H, Zhang M, Peng Y, Zhang C (2018). Gut bacteria selectively promoted by dietary fibers alleviate type 2 diabetes. Science.

[34] Wang X, Yang S, Li S, Zhao L, Hao Y, Qin J, Zhang L, Zhang C et al (2020) Aberrant gut microbiota alters host metabolome and impacts renal failure in humans and rodents. Gut:gutjnl-2019-319766. 10.1136/gutjnl-2019-319766 10.1136/gutjnl-2019-319766 PMC7677483 32241904

[35] Ticinesi A, Nouvenne A, Cerundolo N et al (2019) Gut microbiota, muscle mass and function in aging: a focus on physical frailty and sarcopenia. Nutrients 11. 10.3390/nu11071633 10.3390/nu11071633 PMC6683074 31319564

[36] Nicholson JK, Holmes E, Kinross J, Burcelin R, Gibson G, Jia W, Pettersson S (2012). Host-gut microbiota metabolic interactions. Science.

[37] Barrett E, Ross RP, O'Toole PW, Fitzgerald GF, Stanton C (2012). γ-Aminobutyric acid production by culturable bacteria from the human intestine. J Appl Microbiol.

[38] Holzer P, Farzi A (2014). Neuropeptides and the microbiota-gut-brain axis. Adv Exp Med Biol.

[39] Kawashima K, Misawa H, Moriwaki Y, Fujii YX, Fujii T, Horiuchi Y, Yamada T, Imanaka T, Kamekura M (2007). Ubiquitous expression of acetylcholine and its biological functions in life forms without nervous systems. Life Sci.

[40] Russell WR, Hoyles L, Flint HJ, Dumas ME (2013). Colonic bacterial metabolites and human health. Curr Opin Microbiol.

[41] Landete JM, De las Rivas B, Marcobal A, Muñoz R (2008). Updated molecular knowledge about histamine biosynthesis by bacteria. Crit Rev Food Sci Nutr.

[42] Thomas CM, Hong T, van Pijkeren JP, Hemarajata P, Trinh DV, Hu W, Britton RA, Kalkum M, Versalovic J (2012). Histamine derived from probiotic Lactobacillus reuteri suppresses TNF via modulation of PKA and ERK signaling. PLoS One.

[43] Clarke G, Stilling RM, Kennedy PJ, Stanton C, Cryan JF, Dinan TG (2014). Minireview: Gut microbiota: the neglected endocrine organ. Mol Endocrinol.

[44] Dinan TG, Cryan JF (2017). Gut instincts: microbiota as a key regulator of brain development, ageing and neurodegeneration. J Physiol.

[45] Dalile B, Van Oudenhove L, Vervliet B, Verbeke K (2019). The role of short-chain fatty acids in microbiota-gut-brain communication. Nat Rev Gastroenterol Hepatol.

[46] Samuel BS, Shaito A, Motoike T, Rey FE, Backhed F, Manchester JK, Hammer RE, Williams SC, Crowley J, Yanagisawa M, Gordon JI (2008). Effects of the gut microbiota on host adiposity are modulated by the short-chain fatty-acid binding G protein-coupled receptor, Gpr41. Proc Natl Acad Sci U S A.

[47] Soliman ML, Rosenberger TA (2011). Acetate supplementation increases brain histone acetylation and inhibits histone deacetylase activity and expression. Mol Cell Biochem.

[48] Heck AL, Handa RJ (2019). Sex differences in the hypothalamic-pituitary-adrenal axis response to stress: an important role for gonadal hormones. Neuropsychopharmacology.

[49] Crumeyrolle-Arias M, Jaglin M, Bruneau A, Vancassel S, Cardona A, Daugé V, Naudon L, Rabot S (2014). Absence of the gut microbiota enhances anxiety-like behavior and neuroendocrine response to acute stress in rats. Psychoneuroendocrinology.

[50] Sudo N, Chida Y, Aiba Y, Sonoda J, Oyama N, Yu XN, Kubo C, Koga Y (2004). Postnatal microbial colonization programs the hypothalamic-pituitary-adrenal system for stress response in mice. J Physiol.

[51] Neufeld KM, Kang N, Bienenstock J, Foster JA (2011). Reduced anxiety-like behavior and central neurochemical change in germ-free mice. Neurogastroenterol Motil.

[52] Spielman LJ, Gibson DL, Klegeris A (2018). Unhealthy gut, unhealthy brain: the role of the intestinal microbiota in neurodegenerative diseases. Neurochem Int.

[53] Fülling C, Dinan TG, Cryan JF (2019). Gut microbe to brain signaling: what happens in vagus…. Neuron.

[54] Bonaz B, Bazin T, Pellissier S (2018). The vagus nerve at the interface of the microbiota-gut-brain axis. Front Neurosci.

[55] Osadchiy V, Martin CR, Mayer EA (2019). Gut microbiome and modulation of CNS function. Compr Physiol.

[56] Kaelberer MM, Buchanan KL, Klein ME et al (2018) A gut-brain neural circuit for nutrient sensory transduction. Science 361. 10.1126/science.aat5236 10.1126/science.aat5236 PMC6417812 30237325

[57] Bravo JA, Forsythe P, Chew MV, Escaravage E, Savignac HM, Dinan TG, Bienenstock J, Cryan JF (2011). Ingestion of Lactobacillus strain regulates emotional behavior and central GABA receptor expression in a mouse via the vagus nerve. Proc Natl Acad Sci U S A.

[58] Ogbonnaya ES, Felice D, O'Leary OF (2018). The vagus nerve modulates BDNF expression and neurogenesis in the hippocampus. Eur Neuropsychopharmacol.

[59] Hooper LV, Littman DR, Macpherson AJ (2012). Interactions between the microbiota and the immune system. Science.

[60] Olszak T, An D, Zeissig S, Vera MP, Richter J, Franke A, Glickman JN, Siebert R, Baron RM, Kasper DL, Blumberg RS (2012). Microbial exposure during early life has persistent effects on natural killer T cell function. Science.

[61] Erny D, Hrabě de Angelis AL, Jaitin D, Wieghofer P, Staszewski O, David E, Keren-Shaul H, Mahlakoiv T, Jakobshagen K, Buch T, Schwierzeck V, Utermöhlen O, Chun E, Garrett WS, McCoy KD, Diefenbach A, Staeheli P, Stecher B, Amit I, Prinz M (2015). Host microbiota constantly control maturation and function of microglia in the CNS. Nat Neurosci.

[62] Fung TC, Olson CA, Hsiao EY (2017). Interactions between the microbiota, immune and nervous systems in health and disease. Nat Neurosci.

[63] Cattaneo A, Cattane N, Galluzzi S, Provasi S, Lopizzo N, Festari C, Ferrari C, Guerra UP, Paghera B, Muscio C, Bianchetti A, Volta GD, Turla M, Cotelli MS, Gennuso M, Prelle A, Zanetti O, Lussignoli G, Mirabile D, Bellandi D, Gentile S, Belotti G, Villani D, Harach T, Bolmont T, Padovani A, Boccardi M, Frisoni GB, INDIA-FBP Group (2017). Association of brain amyloidosis with pro-inflammatory gut bacterial taxa and peripheral inflammation markers in cognitively impaired elderly. Neurobiol Aging.

[64] Zhuang ZQ, Shen LL, Li WW, Fu X, Zeng F, Gui L, Lü Y, Cai M, Zhu C, Tan YL, Zheng P, Li HY, Zhu J, Zhou HD, Bu XL, Wang YJ (2018). Gut microbiota is altered in patients with Alzheimer’s disease. J Alzheimers Dis.

[65] Vogt NM, Kerby RL, Dill-McFarland KA (2017). Gut microbiome alterations in Alzheimer’s disease. Sci Rep.

[66] Paley EL, Merkulova-Rainon T, Faynboym A, Shestopalov VI, Aksenoff I (2018). Geographical distribution and diversity of gut microbial NADH:ubiquinone oxidoreductase sequence associated with Alzheimer’s disease. J Alzheimers Dis.

[67] Emery DC, Shoemark DK, Batstone TE, Waterfall CM, Coghill JA, Cerajewska TL, Davies M, West NX, Allen SJ (2017). 16S rRNA next generation sequencing analysis shows bacteria in Alzheimer’s post-mortem brain. Front Aging Neurosci.

[68] Zhang L, Wang Y, Xiayu X, Shi C, Chen W, Song N, Fu X, Zhou R, Xu YF, Huang L, Zhu H, Han Y, Qin C (2017). Altered gut microbiota in a mouse model of Alzheimer’s disease. J Alzheimers Dis.

[69] Bäuerl C, Collado MC, Diaz Cuevas A, Viña J, Pérez Martínez G (2018). Shifts in gut microbiota composition in an APP/PSS1 transgenic mouse model of Alzheimer’s disease during lifespan. Lett Appl Microbiol.

[70] Shen L, Liu L, Ji HF (2017). Alzheimer’s disease histological and behavioral manifestations in transgenic mice correlate with specific gut microbiome state. J Alzheimers Dis.

[71] Honarpisheh P, Reynolds CR, Blasco Conesa MP et al (2020) Dysregulated gut homeostasis observed prior to the accumulation of the brain amyloid-β in Tg2576 mice. Int J Mol Sci 21. 10.3390/ijms21051711 10.3390/ijms21051711 PMC7084806 32138161

[72] Jucker M, Walker LC (2018). Propagation and spread of pathogenic protein assemblies in neurodegenerative diseases. Nat Neurosci.

[73] Del Tredici K, Braak H (2016). Review: Sporadic Parkinson’s disease: development and distribution of α-synuclein pathology. Neuropathol Appl Neurobiol.

[74] Brandscheid C, Schuck F, Reinhardt S, Schäfer KH, Pietrzik CU, Grimm M, Hartmann T, Schwiertz A, Endres K (2017). Altered gut microbiome composition and tryptic activity of the 5xFAD Alzheimer’s mouse model. J Alzheimers Dis.

[75] Semar S, Klotz M, Letiembre M, van Ginneken C, Braun A, Jost V, Bischof M, Lammers WJ, Liu Y, Fassbender K, Wyss-Coray T, Kirchhoff F, Schäfer KH (2013). Changes of the enteric nervous system in amyloid-β protein precursor transgenic mice correlate with disease progression. J Alzheimers Dis.

[76] Ho L, Ono K, Tsuji M, Mazzola P, Singh R, Pasinetti GM (2018). Protective roles of intestinal microbiota derived short chain fatty acids in Alzheimer’s disease-type beta-amyloid neuropathological mechanisms. Expert Rev Neurother.

[77] Gao Q, Wang Y, Wang X (2019). Decreased levels of circulating trimethylamine N-oxide alleviate cognitive and pathological deterioration in transgenic mice: a potential therapeutic approach for Alzheimer’s disease. Aging (Albany NY).

[78] Evin G, Li QX (2012). Platelets and Alzheimer’s disease: potential of APP as a biomarker. World J Psychiatry.

[79] Zhu W, Gregory JC, Org E, Buffa JA, Gupta N, Wang Z, Li L, Fu X, Wu Y, Mehrabian M, Sartor RB, McIntyre TM, Silverstein RL, Tang WHW, DiDonato JA, Brown JM, Lusis AJ, Hazen SL (2016). Gut microbial metabolite TMAO enhances platelet hyperreactivity and thrombosis risk. Cell.

[80] Colciaghi F, Marcello E, Borroni B, Zimmermann M, Caltagirone C, Cattabeni F, Padovani A, di Luca M (2004). Platelet APP, ADAM 10 and BACE alterations in the early stages of Alzheimer disease. Neurology.

[81] Kiriyama Y, Nochi H (2019) The biosynthesis, signaling, and neurological functions of bile acids. Biomolecules 9. 10.3390/biom9060232 10.3390/biom9060232 PMC6628048 31208099

[82] Quinn M, McMillin M, Galindo C, Frampton G, Pae HY, DeMorrow S (2014). Bile acids permeabilize the blood brain barrier after bile duct ligation in rats via Rac1-dependent mechanisms. Dig Liver Dis.

[83] Li T, Chiang JY (2015). Bile acids as metabolic regulators. Curr Opin Gastroenterol.

[84] Gamba P, Testa G, Sottero B, Gargiulo S, Poli G, Leonarduzzi G (2012). The link between altered cholesterol metabolism and Alzheimer’s disease. Ann N Y Acad Sci.

[85] Jia W, Rajani C, Kaddurah-Daouk R, Li H (2019). Expert insights: the potential role of the gut microbiome-bile acid-brain axis in the development and progression of Alzheimer’s disease and hepatic encephalopathy. Med Res Rev.

[86] Zhou Y, Smith D, Leong BJ, Brännström K, Almqvist F, Chapman MR (2012). Promiscuous cross-seeding between bacterial amyloids promotes interspecies biofilms. J Biol Chem.

[87] Schwartz K, Boles BR (2013). Microbial amyloids--functions and interactions within the host. Curr Opin Microbiol.

[88] Du L, Zhang Y, Chen Y, Zhu J, Yang Y, Zhang HL (2017). Role of microglia in neurological disorders and their potentials as a therapeutic target. Mol Neurobiol.

[89] Hansen DV, Hanson JE, Sheng M (2018). Microglia in Alzheimer’s disease. J Cell Biol.

[90] Condello C, Yuan P, Schain A, Grutzendler J (2015). Microglia constitute a barrier that prevents neurotoxic protofibrillar Aβ42 hotspots around plaques. Nat Commun.

[91] Obermeier B, Daneman R, Ransohoff RM (2013). Development, maintenance and disruption of the blood-brain barrier. Nat Med.

[92] Zenaro E, Piacentino G, Constantin G (2017). The blood-brain barrier in Alzheimer’s disease. Neurobiol Dis.

[93] Braniste V, Al-Asmakh M, Kowal C (2014). The gut microbiota influences blood-brain barrier permeability in mice. Sci Transl Med.

[94] Sampson TR, Mazmanian SK (2015). Control of brain development, function, and behavior by the microbiome. Cell Host Microbe.

[95] Hoyles L, Snelling T, Umlai UK, Nicholson JK, Carding SR, Glen RC, McArthur S (2018). Microbiome-host systems interactions: protective effects of propionate upon the blood-brain barrier. Microbiome.

[96] Pellegrini C, Antonioli L, Colucci R, Blandizzi C, Fornai M (2018). Interplay among gut microbiota, intestinal mucosal barrier and enteric neuro-immune system: a common path to neurodegenerative diseases?. Acta Neuropathol.

[97] Itzhaki RF, Lathe R, Balin BJ, Ball MJ, Bearer EL, Braak H, Bullido MJ, Carter C, Clerici M, Cosby SL, del Tredici K, Field H, Fulop T, Grassi C, Griffin WST, Haas J, Hudson AP, Kamer AR, Kell DB, Licastro F, Letenneur L, Lövheim H, Mancuso R, Miklossy J, Otth C, Palamara AT, Perry G, Preston C, Pretorius E, Strandberg T, Tabet N, Taylor-Robinson SD, Whittum-Hudson JA (2016). Microbes and Alzheimer’s disease. J Alzheimers Dis.

[98] Friedland RP (2015). Mechanisms of molecular mimicry involving the microbiota in neurodegeneration. J Alzheimers Dis.

[99] Soscia SJ, Kirby JE, Washicosky KJ, Tucker SM, Ingelsson M, Hyman B, Burton MA, Goldstein LE, Duong S, Tanzi RE, Moir RD (2010). The Alzheimer’s disease-associated amyloid beta-protein is an antimicrobial peptide. PLoS One.

[100] Kumar DK, Choi SH, Washicosky KJ (2016). Amyloid-β peptide protects against microbial infection in mouse and worm models of Alzheimer’s disease. Sci Transl Med.

[101] Eimer WA, Vijaya Kumar DK, Navalpur Shanmugam NK (2018). Alzheimer’s disease-associated β-amyloid is rapidly seeded by Herpesviridae to protect against brain infection. Neuron.

[102] Lukiw WJ (2016). Bacteroides fragilis lipopolysaccharide and inflammatory signaling in Alzheimer’s disease. Front Microbiol.

[103] Liu CY, Wang X, Liu C, Zhang HL (2019). Pharmacological targeting of microglial activation: new therapeutic approach. Front Cell Neurosci.

[104] Zhao Y, Jaber V, Lukiw WJ (2017). Secretory products of the human GI tract microbiome and their potential impact on Alzheimer’s disease (AD): detection of lipopolysaccharide (LPS) in AD hippocampus. Front Cell Infect Microbiol.

[105] Rolls A, Shechter R, London A, Ziv Y, Ronen A, Levy R, Schwartz M (2007). Toll-like receptors modulate adult hippocampal neurogenesis. Nat Cell Biol.

[106] Alexandrov P, Zhai Y, Li W, Lukiw W (2019). Lipopolysaccharide-stimulated, NF-kB-, miRNA-146a- and miRNA-155-mediated molecular-genetic communication between the human gastrointestinal tract microbiome and the brain. Folia Neuropathol.

[107] Hayward JA, Mathur A, Ngo C, Man SM (2018) Cytosolic recognition of microbes and pathogens: inflammasomes in action. Microbiol Mol Biol Rev 82. 10.1128/mmbr.00015-18 10.1128/MMBR.00015-18 PMC6298609 30209070

[108] Halle A, Hornung V, Petzold GC, Stewart CR, Monks BG, Reinheckel T, Fitzgerald KA, Latz E, Moore KJ, Golenbock DT (2008). The NALP3 inflammasome is involved in the innate immune response to amyloid-beta. Nat Immunol.

[109] Arioz BI, Tastan B, Tarakcioglu E, Tufekci KU, Olcum M, Ersoy N, Bagriyanik A, Genc K, Genc S (2019). Melatonin attenuates LPS-induced acute depressive-like behaviors and microglial NLRP3 Inflammasome activation through the SIRT1/Nrf2 pathway. Front Immunol.

[110] Holmes C, Cunningham C, Zotova E, Culliford D, Perry VH (2011). Proinflammatory cytokines, sickness behavior, and Alzheimer disease. Neurology.

[111] Holmes C, Cunningham C, Zotova E, Woolford J, Dean C, Kerr S, Culliford D, Perry VH (2009). Systemic inflammation and disease progression in Alzheimer disease. Neurology.

[112] Sochocka M, Donskow-Łysoniewska K, Diniz BS, Kurpas D, Brzozowska E, Leszek J (2019). The gut microbiome alterations and inflammation-driven pathogenesis of Alzheimer’s disease-a critical review. Mol Neurobiol.

[113] Maynard CL, Elson CO, Hatton RD, Weaver CT (2012). Reciprocal interactions of the intestinal microbiota and immune system. Nature.

[114] Wang X, Sun G, Feng T, Zhang J, Huang X, Wang T, Xie Z, Chu X, Yang J, Wang H, Chang S, Gong Y, Ruan L, Zhang G, Yan S, Lian W, du C, Yang D, Zhang Q, Lin F, Liu J, Zhang H, Ge C, Xiao S, Ding J, Geng M (2019). Sodium oligomannate therapeutically remodels gut microbiota and suppresses gut bacterial amino acids-shaped neuroinflammation to inhibit Alzheimer’s disease progression. Cell Res.

[115] Treangen TJ, Wagner J, Burns MP, Villapol S (2018). Traumatic brain injury in mice induces acute bacterial dysbiosis within the fecal microbiome. Front Immunol.

[116] Sundman MH, Chen NK, Subbian V, Chou YH (2017). The bidirectional gut-brain-microbiota axis as a potential nexus between traumatic brain injury, inflammation, and disease. Brain Behav Immun.

[117] Catania C, Sotiropoulos I, Silva R, Onofri C, Breen KC, Sousa N, Almeida OFX (2009). The amyloidogenic potential and behavioral correlates of stress. Mol Psychiatry.

[118] Niraula A, Sheridan JF, Godbout JP (2017). Microglia priming with aging and stress. Neuropsychopharmacology.

[119] Cryan JF, Dinan TG (2012). Mind-altering microorganisms: the impact of the gut microbiota on brain and behaviour. Nat Rev Neurosci.

[120] Protter DSW, Parker R (2016). Principles and properties of stress granules. Trends Cell Biol.

[121] Wolozin B, Ivanov P (2019). Stress granules and neurodegeneration. Nat Rev Neurosci.

[122] Dobra I, Pankivskyi S, Samsonova A, Pastre D, Hamon L (2018). Relation between stress granules and cytoplasmic protein aggregates linked to neurodegenerative diseases. Curr Neurol Neurosci Rep.

[123] Vanderweyde T, Apicco DJ, Youmans-Kidder K, Ash PEA, Cook C, Lummertz da Rocha E, Jansen-West K, Frame AA, Citro A, Leszyk JD, Ivanov P, Abisambra JF, Steffen M, Li H, Petrucelli L, Wolozin B (2016). Interaction of tau with the RNA-binding protein TIA1 regulates tau pathophysiology and toxicity. Cell Rep.

[124] Ambadipudi S, Biernat J, Riedel D, Mandelkow E, Zweckstetter M (2017). Liquid-liquid phase separation of the microtubule-binding repeats of the Alzheimer-related protein tau. Nat Commun.

[125] Luo J, Mills K, le Cessie S, Noordam R, van Heemst D (2020). Ageing, age-related diseases and oxidative stress: what to do next?. Ageing Res Rev.

[126] Basu M, Courtney SC, Brinton MA (2017). Arsenite-induced stress granule formation is inhibited by elevated levels of reduced glutathione in West Nile virus-infected cells. PLoS Pathog.

[127] Dumitrescu L, Popescu-Olaru I, Cozma L, Tulbă D, Hinescu ME, Ceafalan LC, Gherghiceanu M, Popescu BO (2018). Oxidative stress and the microbiota-gut-brain axis. Oxidative Med Cell Longev.

[128] Kumar A, Wu H, Collier-Hyams LS, Hansen JM, Li T, Yamoah K, Pan ZQ, Jones DP, Neish AS (2007). Commensal bacteria modulate cullin-dependent signaling via generation of reactive oxygen species. EMBO J.

[129] Wang A, Keita ÅV, Phan V, McKay CM, Schoultz I, Lee J, Murphy MP, Fernando M, Ronaghan N, Balce D, Yates R, Dicay M, Beck PL, MacNaughton WK, Söderholm JD, McKay DM (2014). Targeting mitochondria-derived reactive oxygen species to reduce epithelial barrier dysfunction and colitis. Am J Pathol.

[130] Reese AT, Cho EH, Klitzman B et al (2018) Antibiotic-induced changes in the microbiota disrupt redox dynamics in the gut. Elife 7. 10.7554/eLife.35987 10.7554/eLife.35987 PMC6008055 29916366

[131] Cobley JN, Fiorello ML, Bailey DM (2018). 13 reasons why the brain is susceptible to oxidative stress. Redox Biol.

[132] Mottawea W, Chiang CK, Mühlbauer M, Starr AE, Butcher J, Abujamel T, Deeke SA, Brandel A, Zhou H, Shokralla S, Hajibabaei M, Singleton R, Benchimol EI, Jobin C, Mack DR, Figeys D, Stintzi A (2016). Altered intestinal microbiota-host mitochondria crosstalk in new onset Crohn’s disease. Nat Commun.

[133] Ostojic SM (2018). Inadequate production of H(2) by gut microbiota and Parkinson disease. Trends Endocrinol Metab.

[134] Baglietto-Vargas D, Shi J, Yaeger DM, Ager R, LaFerla FM (2016). Diabetes and Alzheimer’s disease crosstalk. Neurosci Biobehav Rev.

[135] Boccardi V, Murasecco I, Mecocci P (2019). Diabetes drugs in the fight against Alzheimer’s disease. Ageing Res Rev.

[136] Steen E, Terry BM, Rivera EJ (2005). Impaired insulin and insulin-like growth factor expression and signaling mechanisms in Alzheimer’s disease--is this type 3 diabetes?. J Alzheimers Dis.

[137] Luchsinger JA, Tang MX, Shea S, Mayeux R (2004). Hyperinsulinemia and risk of Alzheimer disease. Neurology.

[138] Turnbaugh PJ, Ley RE, Mahowald MA, Magrini V, Mardis ER, Gordon JI (2006). An obesity-associated gut microbiome with increased capacity for energy harvest. Nature.

[139] Zarrinpar A, Chaix A, Xu ZZ, Chang MW, Marotz CA, Saghatelian A, Knight R, Panda S (2018). Antibiotic-induced microbiome depletion alters metabolic homeostasis by affecting gut signaling and colonic metabolism. Nat Commun.

[140] Bagarolli RA, Tobar N, Oliveira AG, Araújo TG, Carvalho BM, Rocha GZ, Vecina JF, Calisto K, Guadagnini D, Prada PO, Santos A, Saad STO, Saad MJA (2017). Probiotics modulate gut microbiota and improve insulin sensitivity in DIO mice. J Nutr Biochem.

[141] Soto M, Herzog C, Pacheco JA, Fujisaka S, Bullock K, Clish CB, Kahn CR (2018). Gut microbiota modulate neurobehavior through changes in brain insulin sensitivity and metabolism. Mol Psychiatry.

[142] Bonfili L, Cecarini V, Gogoi O, Berardi S, Scarpona S, Angeletti M, Rossi G, Eleuteri AM (2020). Gut microbiota manipulation through probiotics oral administration restores glucose homeostasis in a mouse model of Alzheimer’s disease. Neurobiol Aging.

[143] Claesson MJ, Jeffery IB, Conde S, Power SE, O’Connor EM, Cusack S, Harris HMB, Coakley M, Lakshminarayanan B, O’Sullivan O, Fitzgerald GF, Deane J, O’Connor M, Harnedy N, O’Connor K, O’Mahony D, van Sinderen D, Wallace M, Brennan L, Stanton C, Marchesi JR, Fitzgerald AP, Shanahan F, Hill C, Ross RP, O’Toole PW (2012). Gut microbiota composition correlates with diet and health in the elderly. Nature.

[144] Berti V, Walters M, Sterling J, Quinn CG, Logue M, Andrews R, Matthews DC, Osorio RS, Pupi A, Vallabhajosula S, Isaacson RS, de Leon MJ, Mosconi L (2018). Mediterranean diet and 3-year Alzheimer brain biomarker changes in middle-aged adults. Neurology.

[145] Del Chierico F, Vernocchi P, Dallapiccola B, Putignani L (2014). Mediterranean diet and health: food effects on gut microbiota and disease control. Int J Mol Sci.

[146] De Filippis F, Pellegrini N, Vannini L (2016). High-level adherence to a Mediterranean diet beneficially impacts the gut microbiota and associated metabolome. Gut.

[147] Shi H, Wang Q, Zheng M, Hao S, Lum JS, Chen X, Huang XF, Yu Y, Zheng K (2020). Supplement of microbiota-accessible carbohydrates prevents neuroinflammation and cognitive decline by improving the gut microbiota-brain axis in diet-induced obese mice. J Neuroinflammation.

[148] Cummings J, Feldman HH, Scheltens P (2019). The “rights” of precision drug development for Alzheimer’s disease. Alzheimers Res Ther.

[149] Mittal R, Debs LH, Patel AP, Nguyen D, Patel K, O'Connor G, Grati M', Mittal J, Yan D, Eshraghi AA, Deo SK, Daunert S, Liu XZ (2017). Neurotransmitters: the critical modulators regulating gut-brain axis. J Cell Physiol.

[150] Anand R, Gill KD, Mahdi AA (2014). Therapeutics of Alzheimer’s disease: past, present and future. Neuropharmacology.

[151] Kandimalla R, Reddy PH (2017). Therapeutics of neurotransmitters in Alzheimer’s disease. J Alzheimers Dis.

[152] Zlomuzica A, Dere D, Binder S, De Souza Silva MA, Huston JP, Dere E (2016). Neuronal histamine and cognitive symptoms in Alzheimer’s disease. Neuropharmacology.

[153] Gibson GR, Hutkins R, Sanders ME, Prescott SL, Reimer RA, Salminen SJ, Scott K, Stanton C, Swanson KS, Cani PD, Verbeke K, Reid G (2017). Expert consensus document: The International Scientific Association for Probiotics and Prebiotics (ISAPP) consensus statement on the definition and scope of prebiotics. Nat Rev Gastroenterol Hepatol.

[154] Suez J, Zmora N, Segal E, Elinav E (2019). The pros, cons, and many unknowns of probiotics. Nat Med.

[155] Abraham D, Feher J, Scuderi GL, Szabo D, Dobolyi A, Cservenak M, Juhasz J, Ligeti B, Pongor S, Gomez-Cabrera MC, Vina J, Higuchi M, Suzuki K, Boldogh I, Radak Z (2019). Exercise and probiotics attenuate the development of Alzheimer’s disease in transgenic mice: role of microbiome. Exp Gerontol.

[156] Rezaei Asl Z, Sepehri G, Salami M (2019). Probiotic treatment improves the impaired spatial cognitive performance and restores synaptic plasticity in an animal model of Alzheimer’s disease. Behav Brain Res.

[157] Yang X, Yu D, Xue L, Li H, Du J (2020). Probiotics modulate the microbiota-gut-brain axis and improve memory deficits in aged SAMP8 mice. Acta Pharm Sin B.

[158] Leblhuber F, Egger M, Schuetz B, Fuchs D (2018). Commentary: Effect of probiotic supplementation on cognitive function and metabolic status in Alzheimer’s disease: a randomized, double-blind and controlled trial. Front Aging Neurosci.

[159] McNulty NP, Yatsunenko T, Hsiao A (2011). The impact of a consortium of fermented milk strains on the gut microbiome of gnotobiotic mice and monozygotic twins. Sci Transl Med.

[160] Sotoudegan F, Daniali M, Hassani S, Nikfar S, Abdollahi M (2019). Reappraisal of probiotics safety in human. Food Chem Toxicol.

[161] Slavin J (2013). Fiber and prebiotics: mechanisms and health benefits. Nutrients.

[162] DeMartino P, Cockburn DW (2020). Resistant starch: impact on the gut microbiome and health. Curr Opin Biotechnol.

[163] Bendiks ZA, Knudsen KEB, Keenan MJ, Marco ML (2020). Conserved and variable responses of the gut microbiome to resistant starch type 2. Nutr Res.

[164] Míguez B, Gómez B, Parajó JC, Alonso JL (2018). Potential of Fructooligosaccharides and xylooligosaccharides as substrates to counteract the undesirable effects of several antibiotics on elder fecal microbiota: a first in vitro approach. J Agric Food Chem.

[165] Schokker D, Fledderus J, Jansen R, Vastenhouw SA, de Bree FM, Smits MA, Jansman AAJM (2018). Supplementation of fructooligosaccharides to suckling piglets affects intestinal microbiota colonization and immune development. J Anim Sci.

[166] Chen D, Yang X, Yang J, Lai G, Yong T, Tang X, Shuai O, Zhou G, Xie Y, Wu Q (2017). Prebiotic effect of fructooligosaccharides from Morinda officinalis on Alzheimer’s disease in rodent models by targeting the microbiota-gut-brain axis. Front Aging Neurosci.

[167] Sun J, Liu S, Ling Z, Wang F, Ling Y, Gong T, Fang N, Ye S, Si J, Liu J (2019). Fructooligosaccharides ameliorating cognitive deficits and neurodegeneration in APP/PS1 transgenic mice through modulating gut microbiota. J Agric Food Chem.

[168] Shokryazdan P, Faseleh Jahromi M, Navidshad B, Liang JB (2017). Effects of prebiotics on immune system and cytokine expression. Med Microbiol Immunol.

[169] Allegretti JR, Kassam Z, Osman M, Budree S, Fischer M, Kelly CR (2018). The 5D framework: a clinical primer for fecal microbiota transplantation to treat Clostridium difficile infection. Gastrointest Endosc.

[170] Allegretti JR, Mullish BH, Kelly C, Fischer M (2019). The evolution of the use of faecal microbiota transplantation and emerging therapeutic indications. Lancet.

[171] Sun J, Xu J, Ling Y, Wang F, Gong T, Yang C, Ye S, Ye K, Wei D, Song Z, Chen D, Liu J (2019). Fecal microbiota transplantation alleviated Alzheimer’s disease-like pathogenesis in APP/PS1 transgenic mice. Transl Psychiatry.

[172] Mullish BH, Quraishi MN, Segal JP, McCune VL, Baxter M, Marsden GL, Moore DJ, Colville A, Bhala N, Iqbal TH, Settle C, Kontkowski G, Hart AL, Hawkey PM, Goldenberg SD, Williams HRT (2018). The use of faecal microbiota transplant as treatment for recurrent or refractory Clostridium difficile infection and other potential indications: Joint British Society of Gastroenterology (BSG) and Healthcare Infection Society (HIS) guidelines. Gut.

